# Supplementary material for: Dissecting the molecular evolution of fluoroquinolone-resistant Shigella sonnei
Source: Nat Commun. 2019 Oct 23;10:4828. doi: 10.1038/s41467-019-12823-0 (PMC6811581; doi:10.1038/s41467-019-12823-0)
Supplement: Supplementary file 1 — Supplementary Information [file 41467_2019_12823_MOESM1_ESM.pdf]

Supplementary Information: Chung The H. *et al.* Dissecting the molecular evolution of fluoroquinolone-resistant *Shigella sonnei*.

Supplementary Figure 1. Population structure and colicin plasmid mapping for 395 CenAsiaIII *Shigella sonnei*. The maximum likelihood phylogeny is identical to that described in Figure 1. The branch colour scheme indicates bootstrap support for the corresponding branch, ranging from low to high (red to black). The rings present the associated data for each taxon, detailed from the innermost to the outermost as follows: (1) population clusters based on Bayesian Analysis of Population Structure (BAPS) hierarchical clustering (Pop1 and Pop2); (2) country (for South and Southeast Asia) or region of original source; (3) presence of colicin plasmid pSSE3; (4) presence of different plasmid Inc types; (5) presence of colicin families. The horizontal scale bar denotes the number of substitutions per site.

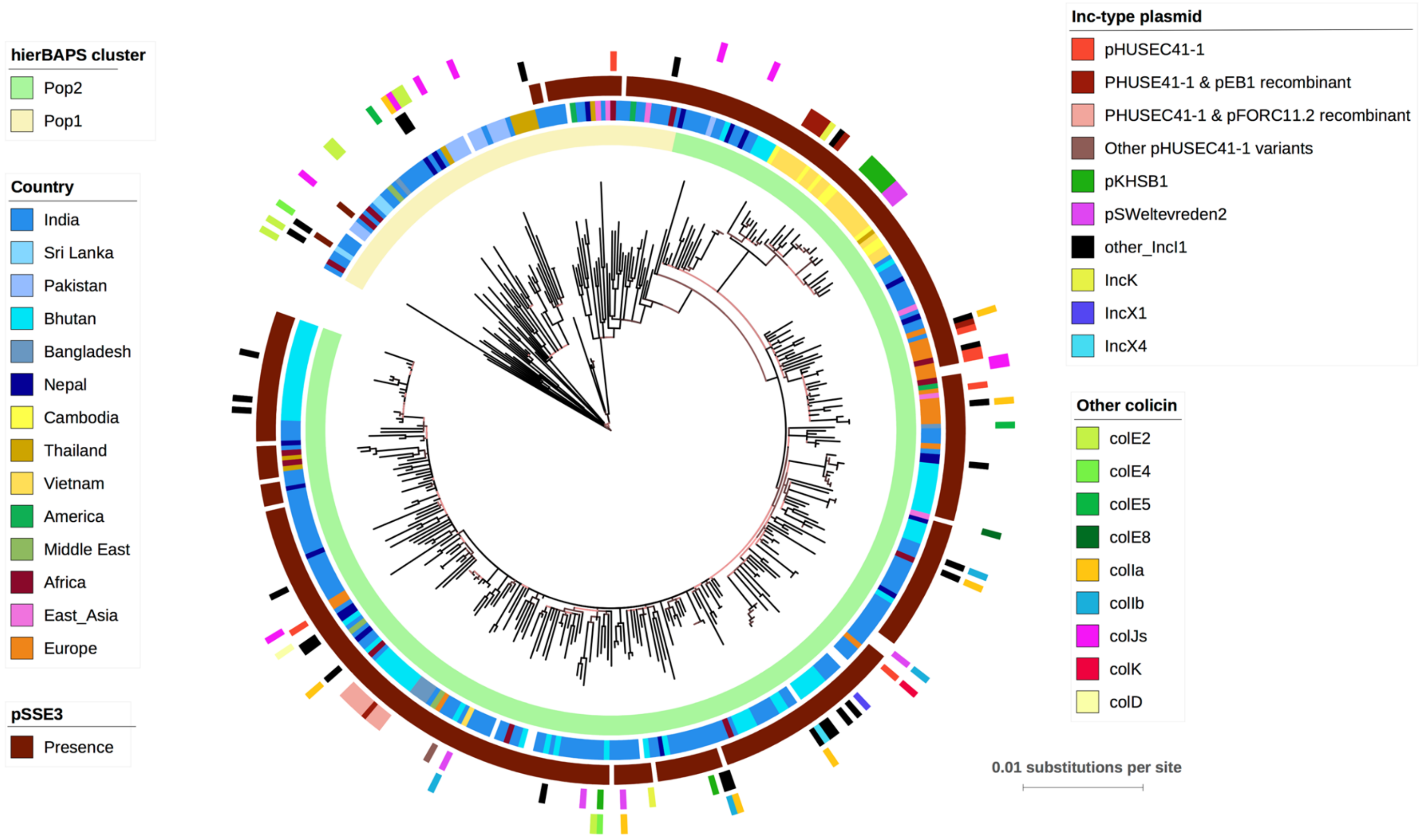

Supplementary Figure 2. Bayesian skyline plot showing the change in effective population size of fluoroquinolone-resistant (FQr) *Shigella sonnei*. The analysis was performed on 134 isolates of FQr *S. sonnei* (all containing *gyrA*-S83L, *parC*-S80I, and *gyrA*-D87G mutations), using the TVM substitution model coupled with a strict molecular clock and Bayesian skyline demographic model.

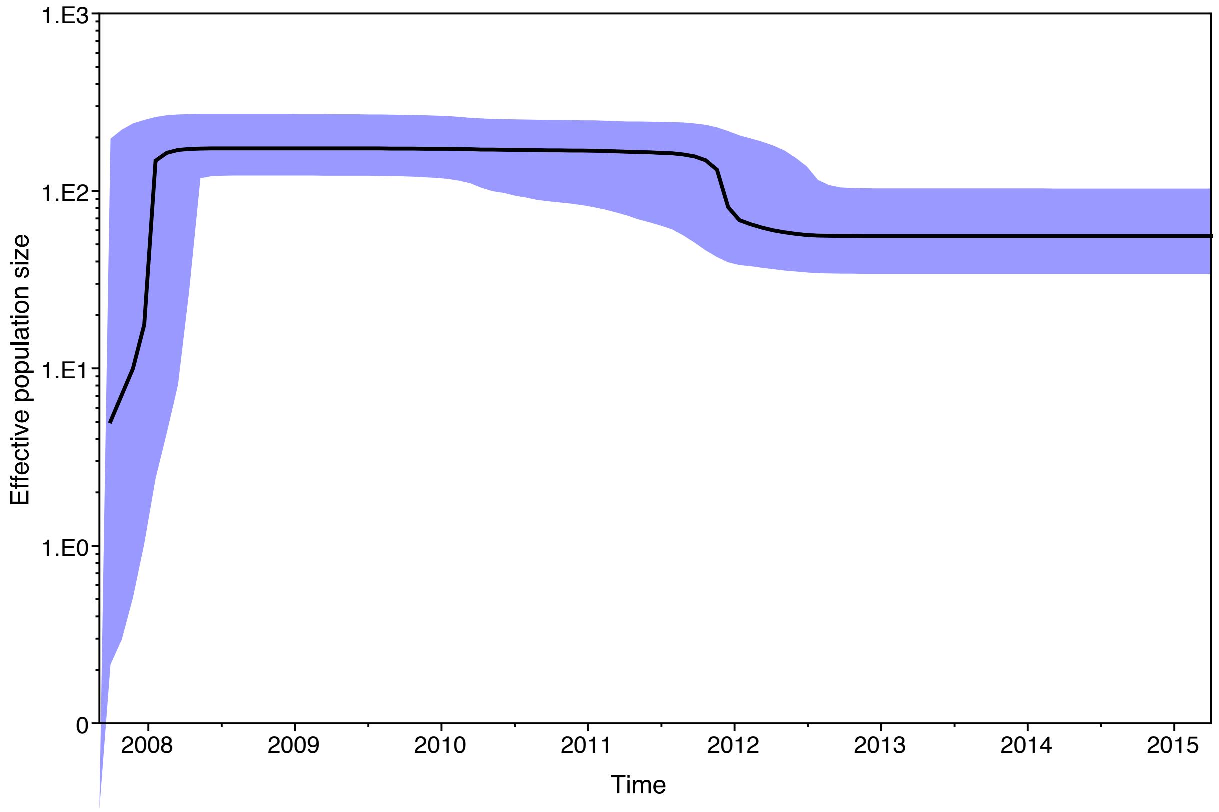

Supplementary Figure 3. Validation of phylogeographical signal in CenAsiaIII *Shigella sonnei.* Stochastic mapping was performed on the original dataset (Figure 3) and ten tip-location randomized datasets (see Materials and Methods). The individual panels compare the inferences made from the original ‘true’ dataset and ten randomizations, measuring the number of transition events from South Asia to Africa, to East Asia/Southeast Asia, and to Europe, as well as the proportional time spent in South Asia during CenAsiaIII’s evolutionary history. The boxplots summarize results of 966 and 500 successful subsamplings of the phylogenies for the original dataset and each randomization, respectively. The asterisks above the median bar within the boxplot of each randomization denote a significant difference between the estimates from the true phylogeny (coloured red) and those derived from the respective randomization (ANOVA Tukey’s test; $p < 0.05$).

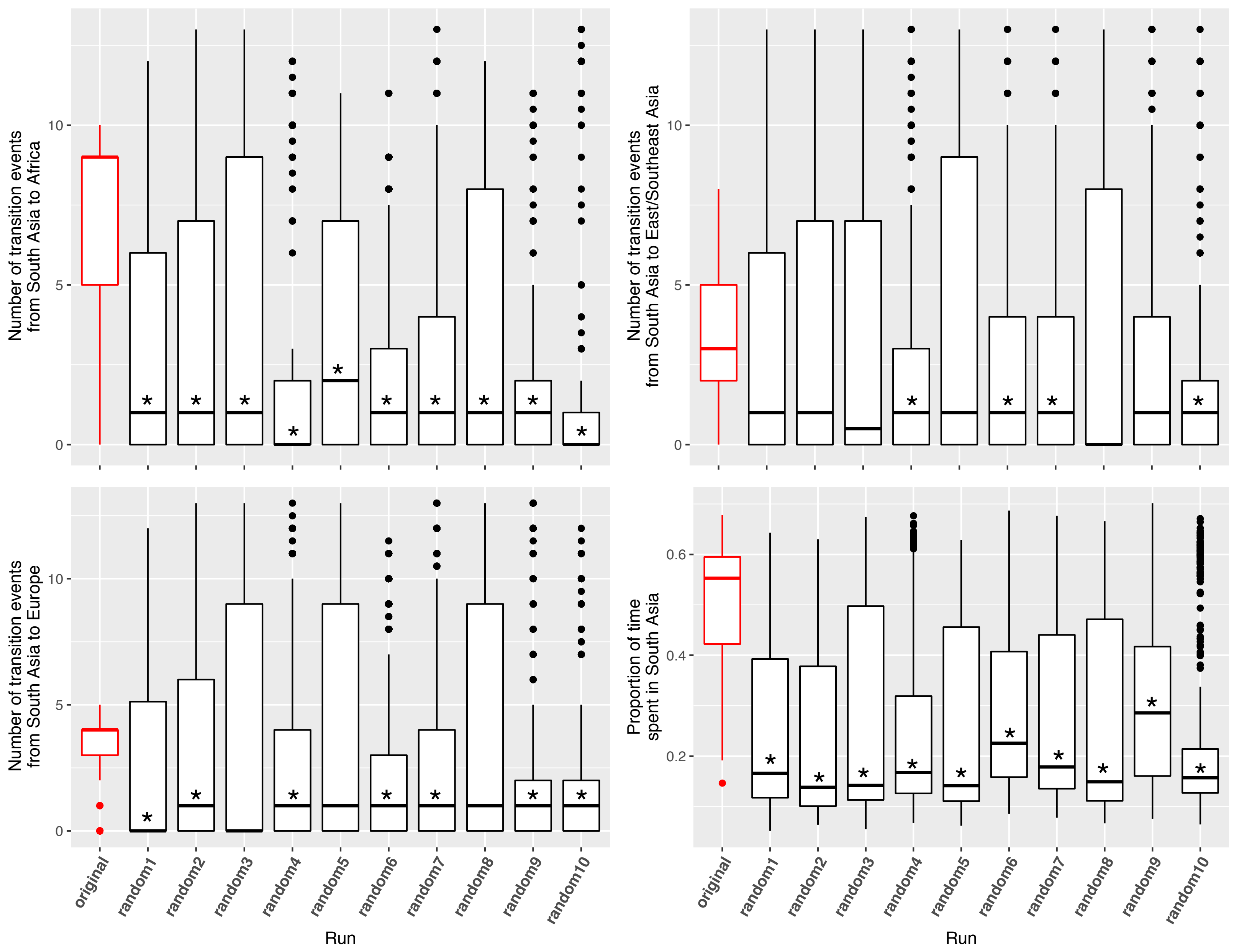

Number of transition events from South Asia to Africa
Number of transition events from South Asia to East/Southeast Asia
Number of transition events from South Asia to Europe
Proportion of time spent in South Asia
0
5
10
0.2
0.4
0.6
original
random1
random2
random3
random4
random5
random6
random7
random8
random9
random10
Run

Supplementary Table 1. List of lineage-defining pseudogenes and gene loss in 395 CenAsiaIII *Shigella sonnei*.

Pseudogene/ gene loss	Mode of inactivation	GO-function	Number of isolates	Estimated time of mutation
ydhV	Disruption by IS600	Putative oxidation-reduction process	395	Since MRCA of CenAsiaIII
asmA	stop codon (loss of 4aa at the end)	regulation of protein targeting to membrane	395	Since MRCA of CenAsiaIII
hofC	stop codon	pilus organization	395	Since MRCA of CenAsiaIII
ybhM	stop codon	Inner membrane	395	Since MRCA of CenAsiaIII
yohL	stop codon	transcription regulation of cobalt and nickel homeostasis	395	Since MRCA of CenAsiaIII
ybeB		regulation of translation	395	Since MRCA of CenAsiaIII
araH	Disruption by IS600	arabinose transport	395	Since MRCA of CenAsiaIII
ydeK	Disruption by IS4	membrane autotransporter	395	Since MRCA of CenAsiaIII
pepE	frameshift	proteolysis	395	Since MRCA of CenAsiaIII
ybeQ	frameshift	oxidative stress response	395	Since MRCA of CenAsiaIII
ybeV	Inframe insertion (+1aa)	Regulation of ATPase activity	395	Since MRCA of CenAsiaIII
yjcR	Disruption by IS1S	sulfur drug transmembrane transport	395	Since MRCA of CenAsiaIII
fliC	Disruption by IS element	Cell motility (flagellin monomer)	395	Since MRCA of CenAsiaIII
nfsA	Disruption by IS1	oxidation-reduction	395	Since MRCA of CenAsiaIII
phoE	Disruption by IS2	transport	341	Since 1996 with gyrA S83L
yeaK	Disruption by IS element	positive regulation of translational fidelity	341	Since 1996 with gyrA S83L
uxaC	stop codon	glucuronate metabolism	341	Since 1996 with gyrA S83L
ybdR	stop codon	oxidation-reduction	341	Since 1996 with gyrA S83L
citF	frameshift	citrate metabolism	341	Since 1996 with gyrA S83L
yqiH	frameshift	pilus organization	341	Since 1996 with gyrA S83L
fliCAZY	Genomic deletion	Flagellum (cell motility)	334	Before parC S80I in 2005 + independent sporadic losses
yddA	Disruption by IS element	transport	309	Before parC S80I in 2005
yaiU	inframe deletion (2aa)	transport	309	Before parC S80I in 2005
flhC	stop codon	regulation of flagellum assembly	20	Bhutanese subclade
ybeM	stop codon	nitrogen compound metabolism	20	Southeast Asian expansion
ecpD	stop codon	pilus organization	20	Southeast Asian expansion
ycbO	stop codon	transport	31	BAPS pop1, since gyrA D87Y mutation

Supplementary Table 2. Distribution of pseudogenization events per gene in 393 CenAsiaIII *Shigella sonnei*.

**Distribution of pseudogenes caused by substitutions and small indels in 393 CenAsiaIII *S. sonnei***									
total number of inactivation mutations	1	2	3	4	5	7	8	14	46
no. of genes	339	34	6	2	1	2	1	1	1
gene names			*hemX*	SSON_0870 (hypothetical protein)	SSON_0348 (hypothetical protein)	SSON_0242 (putative Clp proteinase ATP-binding chain)	*ynfK*	*pepT*	*rpoS*
			mglA	SSON_2732 (phage late control protein)		*puuD*			
			SSON_0868 (putative integrase)						
			yaiU						
			yfeG						
			yggJ						

Supplementary Table 3. Distribution of synonymous and nonsynonymous mutations in 3,637 genes in the CenAsiaIII *Shigella sonnei* population.

**Distribution of mutations in 3637 genes outside the recombination region (as defined in the 'phylogenetic reconstruction' in Methods)**											
no. of nonsynonymous SNPs	0	1	2	3	4	5	6	7	8	10	23
no. of genes	2324	910	284	86	20	6	2	1	2	1	1
gene names								*sigA*	*gyrA, putA*	*mreB*	*rpoS*
no. of synonymous SNPs	0	1	2	3	4	5	6	7			
no. of genes	2763	683	166	22	1	1	0	1			
