# Supplementary material for: Dissecting the molecular evolution of fluoroquinolone-resistant Shigella sonnei
Source: Nat Commun. 2019 Oct 23;10:4828. doi: 10.1038/s41467-019-12823-0 (PMC6811581; doi:10.1038/s41467-019-12823-0)
Supplement: Supplementary file 3 — Description of Additional Supplementary Files [file 41467_2019_12823_MOESM3_ESM.pdf]

Description of Additional Supplementary Files

File Name: Supplementary Data 1

Description: Associated metadata for all Shigella sonnei sequences used in this study.

File Name: Supplementary Data 2

Description: Occurrence of major plasmids found in 395 CenAsiaIII S. sonnei (excluding the S. sonnei large virulence plasmid).
