# Supplementary material for: Dissecting the molecular evolution of fluoroquinolone-resistant Shigella sonnei
Source: Nat Commun. 2019 Oct 23;10:4828. doi: 10.1038/s41467-019-12823-0 (PMC6811581; doi:10.1038/s41467-019-12823-0)
Supplement: Supplementary file 6 — Reporting Summary [file 41467_2019_12823_MOESM6_ESM.pdf]

nature research

Corresponding author(s): Maia A. Rabaa

Last updated by author(s): Sep 13, 2019

Reporting Summary

Nature Research wishes to improve the reproducibility of the work that we publish. This form provides structure for consistency and transparency in reporting. For further information on Nature Research policies, see Authors & Referees and the Editorial Policy Checklist.

Statistics

For all statistical analyses, confirm that the following items are present in the figure legend, table legend, main text, or Methods section.

n/a	Confirmed	
☐	☒	The exact sample size (*n*) for each experimental group/condition, given as a discrete number and unit of measurement
☐	☒	A statement on whether measurements were taken from distinct samples or whether the same sample was measured repeatedly
☐	☒	The statistical test(s) used AND whether they are one- or two-sided<br>*Only common tests should be described solely by name; describe more complex techniques in the Methods section.*
☒	☐	A description of all covariates tested
☒	☐	A description of any assumptions or corrections, such as tests of normality and adjustment for multiple comparisons
☒	☐	A full description of the statistical parameters including central tendency (e.g. means) or other basic estimates (e.g. regression coefficient) AND variation (e.g. standard deviation) or associated estimates of uncertainty (e.g. confidence intervals)
☐	☒	For null hypothesis testing, the test statistic (e.g. *F*, *t*, *r*) with confidence intervals, effect sizes, degrees of freedom and *P* value noted<br>*Give P values as exact values whenever suitable.*
☐	☒	For Bayesian analysis, information on the choice of priors and Markov chain Monte Carlo settings
☒	☐	For hierarchical and complex designs, identification of the appropriate level for tests and full reporting of outcomes
☒	☐	Estimates of effect sizes (e.g. Cohen's *d*, Pearson's *r*), indicating how they were calculated

Our web collection on statistics for biologists contains articles on many of the points above.

Software and code

Policy information about availability of computer code

Data collection	Illumina Sequencing Platform (HiSeq, MiSeq, NextSeq)
Data analysis	MS Excel, R, RAxML, BEAST, GATK

For manuscripts utilizing custom algorithms or software that are central to the research but not yet described in published literature, software must be made available to editors/reviewers. We strongly encourage code deposition in a community repository (e.g. GitHub). See the Nature Research guidelines for submitting code & software for further information.

Data

Policy information about availability of data

All manuscripts must include a data availability statement. This statement should provide the following information, where applicable:

- Accession codes, unique identifiers, or web links for publicly available datasets
- A list of figures that have associated raw data
- A description of any restrictions on data availability

The authors declare that the data supporting the findings of this study are available within the paper and its Supplementary Information files, and, also available from the corresponding author upon reasonable request.

Field-specific reporting

Please select the one below that is the best fit for your research. If you are not sure, read the appropriate sections before making your selection.

☒ Life sciences ☐ Behavioural & social sciences ☐ Ecological, evolutionary & environmental sciences

For a reference copy of the document with all sections, see nature.com/documents/nr-reporting-summary-flat.pdf

Life sciences study design

All studies must disclose on these points even when the disclosure is negative.

Sample size	No statistical analysis was carried out.
Data exclusions	Two sequences with poor mapping quality were excluded.
Replication	All experiments were successfully replicated at least twice.
Randomization	Not applicable.
Blinding	Not applicable.

Reporting for specific materials, systems and methods

We require information from authors about some types of materials, experimental systems and methods used in many studies. Here, indicate whether each material, system or method listed is relevant to your study. If you are not sure if a list item applies to your research, read the appropriate section before selecting a response.

Materials & experimental systems

n/a	Involved in the study	
☒	☐	Antibodies
☒	☐	Eukaryotic cell lines
☒	☐	Palaeontology
☒	☐	Animals and other organisms
☐	☒	Human research participants
☒	☐	Clinical data

Methods

n/a	Involved in the study	
☒	☐	ChIP-seq
☒	☐	Flow cytometry
☒	☐	MRI-based neuroimaging

Human research participants

Policy information about studies involving human research participants

Population characteristics	N/A (clinical data not used in this study)
Recruitment	S. sonnei isolates from Bhutan, Thailand, and Vietnam were collected during diarrheal surveillance studies. The target patient groups for these studies were generally hospitalised children aged less than 5 y residing in close proximity to the study centres. S. sonnei isolates from Cambodia were collected at the Angkor Hospital for Children (AHC) in Siem Reap province from the routine diagnostic laboratory; no patient data were collected. The ciprofloxacin-resistant S. sonnei from countries outside Asia were collected and characterized by the National Salmonella, Shigella, and Listeria monocytogenes Reference Laboratory, Galway, Ireland, and the Microbiological Diagnostic Unit Public Health Laboratory, Melbourne, Australia. These isolates were generally, but not exclusively, obtained from patients reporting recent travel to countries with a high incidence of shigellosis in Asia.
Ethics oversight	IRB approval was granted for these studies (including organism characterization) from the Research Ethics Board of Health (REBH), Ministry of Health, Bhutan (Bhutan study), the Walter Reed Army Institute of Research (WRAIR) Institutional Review Board, USA (Bhutan and Thailand studies), the Oxford Tropical Research Ethics Committee (OxTREC), UK, and the Hospital for Tropical Diseases Ho Chi Minh City, Vietnam (Vietnam study). Written (Vietnam) or oral (Thailand and Bhutan) consent was obtained from a parent or guardian at the time of enrolment into the study. S. sonnei isolates from Cambodia were collected at the Angkor Hospital for Children (AHC) in Siem Reap province from the routine diagnostic laboratory; no patient data were collected, and these organisms were not subject to local or international IRB approval. The ciprofloxacin-resistant S. sonnei from countries outside Asia were collected and characterized by the National Salmonella, Shigella, and Listeria monocytogenes Reference Laboratory, Galway, Ireland, and the Microbiological Diagnostic Unit Public Health Laboratory, Melbourne, Australia. These isolates were generally, but not exclusively, obtained from patients reporting recent travel to countries with a high incidence of shigellosis in Asia. As these isolates were from anonymous sources and collected at local public health laboratories, these were not subject to IRB approval, and informed consent was not required.

Note that full information on the approval of the study protocol must also be provided in the manuscript.
